# Defense Strategies: The Role of Transcription Factors in Tomato–Pathogen Interaction

**DOI:** 10.3390/biology11020235

**Published:** 2022-02-01

**Authors:** Maria Doroteia Campos, Maria do Rosário Félix, Mariana Patanita, Patrick Materatski, André Albuquerque, Joana A. Ribeiro, Carla Varanda

**Affiliations:** 1 MED—Mediterranean Institute for Agriculture, Environment and Development, Instituto de Investigação e Formação Avançada, Universidade de Évora, Pólo da Mitra, Ap. 94, 7006-554 Évora, Portugal; mpatanita@uevora.pt (M.P.); pmateratski@uevora.pt (P.M.); andrealb@uevora.pt (A.A.); joanaar@uevora.pt (J.A.R.); carlavaranda@uevora.pt (C.V.); 2 MED—Mediterranean Institute for Agriculture, Environment and Development & Departamento de Fitotecnia, Escola de Ciências e Tecnologia, Universidade de Évora, Pólo da Mitra, Ap. 94, 7006-554 Évora, Portugal; mrff@uevora.pt

**Keywords:** *Solanum lycopersicum*, transcription factors, defense mechanisms, disease resistance, biotic stress

## Abstract

**Simple Summary:**

Tomato is one of the most cultivated and economically important vegetable crops throughout the world. It is affected by a panoply of different pathogens that cause infectious diseases that reduce tomato yield and affect product quality, with the most common symptoms being wilts, leaf spots/blights, fruit spots, and rots. To survive, tomato, as other plants, have developed elaborate defense mechanisms against plant pathogens. Among several genes already identified in tomato response to pathogens, we highlight those encoding the transcription factors (TFs). TFs are regulators of gene expression and are involved in large-scale biological phenomena. Here, we present an overview of recent studies of tomato TFs regarding defense responses to pathogen attack, selected for their abundance, importance, and availability of functionally well-characterized members. Tomato TFs’ roles and the possibilities related to their use for genetic engineering in view of crop breeding are presented.

**Abstract:**

Tomato, one of the most cultivated and economically important vegetable crops throughout the world, is affected by a panoply of different pathogens that reduce yield and affect product quality. The study of tomato–pathogen system arises as an ideal system for better understanding the molecular mechanisms underlying disease resistance, offering an opportunity of improving yield and quality of the products. Among several genes already identified in tomato response to pathogens, we highlight those encoding the transcription factors (TFs). TFs act as transcriptional activators or repressors of gene expression and are involved in large-scale biological phenomena. They are key regulators of central components of plant innate immune system and basal defense in diverse biological processes, including defense responses to pathogens. Here, we present an overview of recent studies of tomato TFs regarding defense responses to biotic stresses. Hence, we focus on different families of TFs, selected for their abundance, importance, and availability of functionally well-characterized members in response to pathogen attack. Tomato TFs’ roles and possibilities related to their use for engineering pathogen resistance in tomato are presented. With this review, we intend to provide new insights into the regulation of tomato defense mechanisms against invading pathogens in view of plant breeding.

## 1. Introduction

Plant pathogens cause severe losses in agriculture systems in terms of economics and production and are increasing worldwide. Although many plant pathogens are well known, new virulent strains, pathotypes, or races, together with new emerging pathogens, have had a negative impact in the production potential of agriculture [1]. Additionally, climate change has an impact on disease incidence and severity and on the geographic distribution of plant pathogens, with consequences for agricultural production, turning challenging the plant disease management [2].

**Figure 1:**
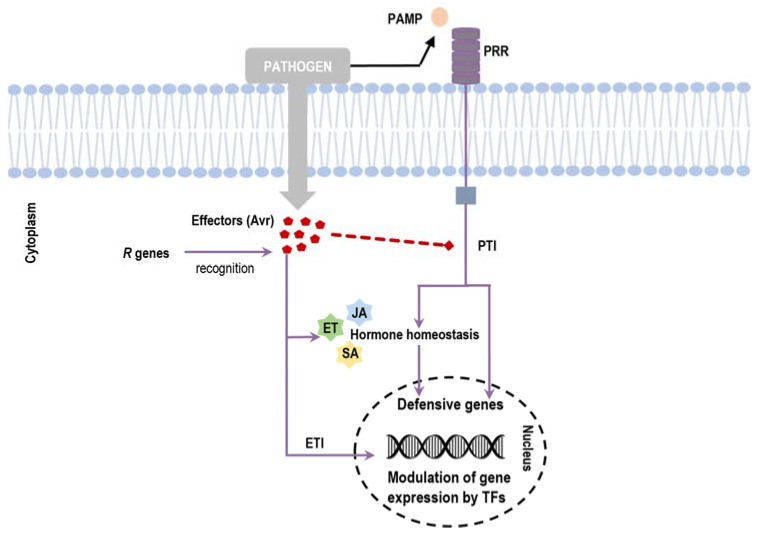
Recognition of a pathogen by the plant and induction of active immune response. Pathogen-associated molecular patterns (PAMPs) are perceived by plant transmembrane pattern recognition receptors (PRRs), which induce signaling cascades and lead to PAMP triggered immunity (PTI). During pathogen attack, pathogens produce effectors molecules (Avr) to increase their pathogenicity into the host cells to suppress PTI and to interfere with hormonal balance. Plants having corresponding resistance (R) genes recognize effectors and activate immune responses (effector-triggered immunity, ETI). After recognition, it is triggered the transcription of the cascade of plant defense mechanisms, and the activation of genes to a robust and quick defense response is induced. ETI response may include altering chromatin configuration that further facilitates access by transcription factors (TFs). Adapted from [10].

Plant pathogens mostly comprise viruses, bacteria, fungi, and nematodes that cause symptoms on leaves, stems, roots, vascular systems, and fruits [3,4,5]. Due to their sedentary nature, plants sense the stress signals and, for their adaptation and survival, it is essential that they give appropriate responses [6]. To survive, plants have developed highly sophisticated defense mechanisms against pathogens [7]. During the pathogen attack, conserved molecular patterns are recognized by the plants (pathogen-associated molecular patterns, PAMPs) at the pathogen cell surface and trigger basal immune responses (PAMP-triggered immunity, PTI). These two-way efficient communication strategies form the plant innate immune system [8,9], playing relevant functions through signaling transduction pathways in order to modulate regulatory proteins (e.g., transcription factors (TFs) and protein kinases) and pathogenesis-related proteins (PR) [6,10]. When the interaction between a plant and a plant pathogen occurs and causes infection, it is designated as compatible interaction. Some pathogens secrete effectors to increase their pathogenicity into the host cells to suppress PTI; this prompt plants with the corresponding resistance (R) proteins to directly or indirectly recognize the effectors and initiate immune responses named effector-triggered immunity (ETI), such as the hypersensitive response (HR) [11,12]. Signal transduction and the fine-tuning of gene expression are requisites to regulate these defense mechanisms in PTI and ETI [6,13,14]. Figure 1 schematizes the regulatory pathways induced in plants upon pathogen infection.

The modulation of gene transcription is an essential step for an efficient defense response in host cells. Transcriptional re-programming of the plant cell involves considerable changes in gene expression to support the plant defense rather than other cellular processes such as development and growth [6]. It was suggested that plants may recognize each attacking pathogen specifically and have a distinct transcriptional response to different pathogens [15].

Transcription factors (TFs) are central components of plant defense signaling and adaptation mechanisms. They are DNA-binding proteins that play roles in the modulation of gene expression by binding to transcriptional regulatory regions called *cis*-elements in the gene promoters [16,17], specifically activating or repressing expression of target genes and directing the expression in a synchronized manner [10]. A collection of similar DNA sequences is recognized by each TF, which can be represented as binding site motifs. Motifs’ characterization is a crucial step for a better understanding of the regulatory functions of TFs that consequently shape gene regulatory networks [16]. Major TF families are crucial regulators of various genes related to the response to different stresses [18]. After induction by pathogen attack, PR genes are activated and/or HR response is promoted by TFs [10].

A deeper knowledge of the molecular mechanisms that involve the relation between a plant and a specific pathogen has been highly facilitated by the technological developments that occurred over the last decade. Transcriptome analyses turns possible the discovery of the molecular basis of plant–pathogen interaction, allowing the scrutiny of the molecular repertoires available for defense responses in host plants [19], including the identification of genes coding for TFs [19,20]. In addition, there were already performed studies that functionally characterized TFs genes in several plant species, in order to enhance their resistance against several stress situations, in view of crop improvement [18].

Tomato (*Solanum lycopersicum*) has been deeply studied, showing high interest as a model plant species [21]. It is affected by an abundance of pathogens that cause serious diseases, hence reducing yield and affecting product quality [22] and, contrary to other model organisms, tomato has important agronomic characteristics, as the production of fleshy fruits, widely used in human diet [21].

With diverse germplasms available across the world, breeding programs have made great strides in tomato improvement, with many morphological distinct cultivars developed from the single species of *S. lycopersicum*. However, the cultivated *S. lycopersicum* species was estimated to contain only about 5% of the total genetic variation existing in all tomato species, which occurred during its domestication and early breeding [23]. Unfortunately, resistance to important traits such as biotic and abiotic factors have been impaired during the process of domestication.

To compensate for the limited genetic diversity within the cultivated *S. lycopersicum* species, the genetic engineering that involves the transfer of desired genes broadens the chances for crop improvement. Several genome editing approaches for breeding goals applications were already implemented for tomato resistance to various biotic and abiotic stresses and for traits improvement (see review in [24]).

**Figure 2:**
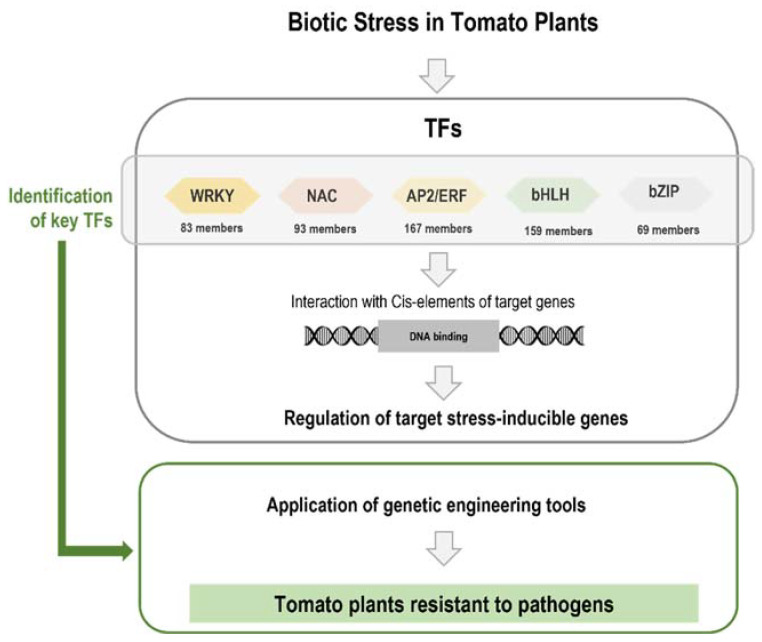
Tomato transcription factors (TFs) as key components of the regulation of target gene expression in response to biotic stress and their use for engineering pathogen resistance in tomato plants.

In our previous review [19], we reported studies on tomato transcriptome profiling regarding differential gene expression in response to pathogens. We have identified genes encoding TFs as commonly differentially expressed regardless of the pathogens’ type: bacteria, fungi, oomycetes, viruses, or nematodes. Since TFs are key components of plant defense mechanisms and therefore excellent candidates regarding crop improvement, in the present review, we focus on the identification and on the role of different families of TFs on tomato response to a large range of plant pathogens, including studies for engineering pathogen resistance in tomato plants. Figure 2 demonstrates a schematic representation of the approach followed in the present work. 

## 2. Genome Editing to Increase Tomato Resistance to Biotic Stress

A high percentage of crops become deteriorated annually all over the world during growth or post-harvest storage due to diseases caused by several types of pathogens, mainly fungi, bacteria, viruses, and nematodes. Tomato, one of the most important vegetable crops worldwide, had an estimated production near 190 million tonnes in 2020 (https://www.fao.org/faostat/en/#data/QCL/visualize, accessed on 27 January 2022). The quality and yield of tomato is highly reduced by infectious diseases, and the most common disease symptoms consisting of fruit spots, rots, wilts, and leaf spots/blights [22].

Over the last 50 years, the prevailing control measure for disease management in crop production has been through the application of chemical pesticides, with the continuous exposure adversely affecting the soil texture, productivity, water contamination, and nutritional content of vegetables, as well as human health [25]. In this context, novel emerging technologies are gaining importance for a better agricultural sustainability. Traditionally, breeding applied to plant species has allowed the generation of new crop varieties, but more recently, new technologies with the precise introduction of desirable alleles into many different, locally adapted elite varieties offer an opportunity to rapidly generate improved varieties with reduced costs [26]. Plant molecular biology and biotechnology studies have supported plant defense strategies, making possible the selection of traits that might decrease pathogen’s aggression [27]. Genetic transformation, offering an opportunity to stably insert specific gene sequences into a host plant, remains generally the most frequently exploited strategy [28]. Biotechnological tools can, for example, counterbalance pathogen aggressiveness and consequent yield losses, through the overexpression of defense genes against crop pathogens [27] and more recently with new breeding techniques that have been developed and optimized. Discussions are ongoing concerning ethical and societal questions regarding the definition of genetic modification, and protection of biological diversity from the potential risks posed by living modified organisms resulting from modern biotechnology for mankind and environment defined in the Cartagena Protocol on Biosafety [29].

Genome editing is a great innovation in plant breeding that facilitates efficient, precise, and targeted modifications at genomic loci that will allow the obtention of transgene-free plants. These plants are identical or similar to the ones generated by conventional breeding techniques, with the genome editing enabling the precise editing of a gene of interest [30]. Over recent years, many advances have been made in the RNA-based gene regulation approach, i.e., RNA interference (RNAi). RNAi is a gene-silencing phenomenon, which can be used for the development of crops that are tolerant to stress conditions and disease-resistant, not only by the modification of the expression of a gene but also for the assessment of gene function and plant metabolic engineering [31]. Gene silencing occurs through transcriptional gene silencing (TGS) or post-transcriptional gene silencing (PTGS). Gene silencing may be induced by viruses—virus-induced gene silencing (VIGS)—a tool in PTGS for the functional characterization of genes in plants that has been widely used in several plant species [32]. Gene-editing technologies such as the ones based on clustered regularly interspaced short palindromic repeats, (CRISPR)/CRISPR-associated protein (CRISPR/Cas), are powerful tools for precise targeted modifications of nearly all crops’ genome sequences to generate variation and accelerate breeding efforts. CRISPR/Cas allows targeting a sequence for gene knockout, knock in, and replacement, along with observing and regulating gene expression by binding a specific sequence at the genome and epigenome levels [33]. Tools for editing the genome have already been applied for tomato breeding to increase the resistance to biotic stresses (see review in [24,34,35]). As an example, we report the silencing of the *Powdery Mildew Resistance 4* (*PMR4*) gene through RNAi that resulted in resistance to the tomato powdery mildew fungus *Oidium neolycopersici* [36]. Furthermore, a non-transgenic tomato variety also resistant to the same fungus was generated using the CRISPR/Cas9 technology through an edited homozygous loss-of-function mutations of *MILDEW RESISTANT LOCUS O* (*slmlo1*) tomato variety [26].

## 3. Transcription Factors Are Involved in Plant Defense Response

Several families of TFs have been found according to specific amino acid sequences and conserved DNA-binding domains [18]. The role of TFs on transcriptional reprogramming, as transcriptional activators or repressors, leads to their involvement in large-scale biological phenomena that include growth and development [17,18]. In plants, the successful defense response is dependent on the precise and on-time detection of the pathogenic agent and ensuing induction of the responsible pathways to move away the pathogens [12]. The plant defense response is achieved by the key role that TFs have on transcriptional reprogramming, involving an infinitive of highly synchronized complexed molecular, biochemical, and physiological changes [12]. The roles of TFs in plant defense have been highlighted by several authors (see review in [10]). Figure 1 and Figure 2 broadly summarize the general activation of TFs in response to pathogen attack.

TFs involved in the various defense pathways with critical roles in immune responses against pathogens mostly belong to the families WRKY, NAC (NAM, ATAF, and CUC), AP2/ERF (Apetala2/Ethylene Responsive Factor), bZIP (basic leucine zipper domain), and bHLH (basic helix-loop-helix). Below, we briefly describe the structural and functional aspects of those families.

### 3.1. WRKY

WRKY TFs compose one of the largest families of transcriptional regulators [37]. They consist of ~60 amino acids, with the highly conserved WRKYGQK domain at the N-terminus and a zinc-finger motif at the C-terminus. WRKY TFs can be divided into Groups I, II, and III, based on the type of zinc-finger structures and the number of WRKY domains [38]. In general, target genes are regulated by WRKY TFs through binding W-box (TTGACY, with the core sequence TGAC) *cis*-elements in gene promoters, although other binding sites have been reported [38,39]. The complete genome sequencing of many plants has resulted in a more comprehensive identification of multiple members of the WRKY TF class, with several genes found in several plant species [10,40,41,42,43].

The involvement of WRKYs in PTI and ETI takes place at different regulatory levels [44]. They can interact with PAMPs or effector proteins to activate or repress both PTI and ETI. Insights on the involvement of WRKYs in different aspects of plant biology are given through numerous expression and functional studies. They are involved in several processes such as seed dormancy, germination, and development, besides abiotic and biotic stress responses [37]. Several *WRKY* genes are responsive to pathogens, elicitors, and defense-related phytohormones (i.e. salicylic acid (SA) and jasmonic acid (JA)), which implies a major role of this family in plant immunity [38].

### 3.2. NAC

The NAC (NAM, CUC, and ATAF) gene family belongs to a larger family encoding plant-specific TFs [45]. The members of this family are widespread in plants and are characterized by the presence of a highly conserved N-terminal region, known as NAC domain (~150 amino acids). This region functions as a DNA-binding domain and is also responsible for oligomerization into dimer [39,46]. However, their C-terminal transcription regulatory domains vary and can activate or repress transcription, with conserved specific motifs for a given subgroup within NAC subfamilies [44,46,47].

*NAC* genes have been identified through genome-wide studies and expression analyses in different plant species such as rice, tomato, tobacco, or cucumber [48,49,50,51]. Immunity-related NAC TFs, belonging to different NAC subfamilies, have been reported to play important roles in plant immunity as negative or positive regulators, modulators of HR, and stomatal immunity or targets of pathogen effectors (see review in [52]). *NAC* genes expression is induced by abiotic [53] and biotic stresses, with genes involved in defense response against pathogen invasion, insect feeding, and wounding [54].

### 3.3. AP2/ERF

The AP2/ERF gene family is an important family encoding plant-specific TFs, with members identified in many plant species [55,56]. This superfamily is defined by the AP2/ERF domain, constituted by 60 to 70 amino acids. The AP2/ERF domain is involved in DNA binding, and contains an N-terminal, a three-stranded β-sheet, and a C-terminal α-helix [57]. It is divided into different sub-families: AP2 (APETALA2), ERF (ethylene responsive factors), RAV (Related to ABI3/VP), DREB (dehydration responsive element binding), and soloist [55,58]. AP2/ERF TFs have been demonstrated to play important roles in developmental processes, tolerance to biotic and abiotic stresses, and hormone signaling transduction in plants [56,59,60,61,62]. *AP2/ERF*, as the final responsive genes in the ethylene signaling pathway, have a role on the modulation of phytohormone biosynthesis, including ethylene, auxin, cytokinin, gibberellin, ABA, and jasmonate (see review in [62]).

### 3.4. bHLH

bHLH proteins have been widely studied in plants, including *Capsicum annum*, tobacco, potato, and tomato [63,64,65,66], although they are distributed in eukaryotes [64]. The family members of this large superfamily of TFs are divided into several groups and contain a bHLH domain that comprises approximately 60 amino acids, including a basic region and an HLH region with several functions [17,67]. The basic region, characterized by approximately 17 amino acids located at the N-terminus of the domain, is a DNA-binding region that allows HLH proteins to bind to a consensus hexanucleotide E-box (CANNTG) [64,68]. bHLH TFs have a central role in many physiological, metabolic, and developmental processes in higher organisms [66] and are associated to the plants primary and specialized metabolites [63]. Some of them are closely related to hormone signaling, phytochrome signaling, flavonoid biosynthesis, and stress responses including immunity against pathogenic agents such as fungi and bacteria [69,70,71].

### 3.5. bZIP

bZIP proteins belong to a large family of plant TFs and are divided into several groups. This family is composed by a bZIP domain, consisting of 60 to 80 amino acids, a DNA-binding basic region, and a leucine zipper for homo- or hetero-dimerization [17]. It was already demonstrated by genetic and molecular studies that bZIP factors in plants regulate diverse biological processes that include seed formation, floral development, and also responses to abiotic and biotic stresses [17].

*bZIP* genes have been identified in all eukaryotes including plant species such as *Arabidopsis*, maize, pepper, and tomato [72,73,74,75]. Amongst the bZIP TFs, the well-studied TGA proteins play a central role in signaling mediated by SA and in defense against pathogen attack [10]. The involvement of bZIP TFs in plant defense was already proven in plants such as *Arabidopsis*, in which two of the 10 groups of bZIP TFs were shown to play a role in plant innate immunity [14], and in tobacco and maize, in which some group members are proposed to participate in defense response [73,76]. Additionally, a *bZIP* gene is highly expressed in pepper plants after inoculation with the biotrophic bacteria *Xanthomonas campestris* pv. *vesicatoria* and by defense-related hormones such as ethylene, methyl jasmonate, and SA [77]. bZIP TFs from the TGA family regulate the PR genes due to their physical interaction with the identified positive regulator, nonexpresser of PR gene1 (NPR1), as reported by Kesarwani et al. [76].

## 4. Transcription Factors Are Involved in Tomato Resistance to Biotic Stresses

The tomato genome was completely sequenced [78], and the freely available genome database provides an excellent platform, offering an opportunity to characterize gene families, including the TFs at the genome-wide level. Figure 2 and Table S1 indicate the number of tomato TFs belonging to the different families and subfamilies. Tomato is, as described above, susceptible to several diseases caused by a wide range of pathogens. Novel methodologies have allowed a deeper knowledge on the molecular mechanisms involved in tomato–pathogen interaction and identify TFs as excellent candidates for crop breeding [19].

Genome-editing tools for improving traits such as disease resistance have demonstrated their relevance in tomato response to pathogen infection [24]. Below, we describe studies on the identification of TFs in tomato and report relevant research on the involvement of the different TFs’ families and genes on the response of tomato to infection by several pathogens (Table 1). We also identify genetic engineering tools to incorporate new sources of resistance in tomato (Table 1). Although studies on the involvement of genes coding for tomato TFs in response to biotic stress performed in other species such as *Arabidopsis* or rice (i.e., [40]) can be found, they are not being considered in the present study.

A total of 83 *WRKY* genes were identified in tomato [96] (Table S1), and several studies demonstrate their roles in tomato defense by showing altered expression of *WRKYs* genes upon infection of pathogens, as well as research involving overexpression and/or silencing of different *WRKYs* genes (Table 1).

Liu and co-authors [79] identified a responsive *WRKY* gene *SlDRW1* (*S. lycopersicum* defense-related *WRKY1*), whose expression was significantly induced by *Botrytis cinerea*. Silencing of *SlDRW1* resulted in increased severity of disease caused by *B. cinerea*, attenuating the defense response and affecting the expression of a group of genes involved in defense response. Interestingly, the overexpression in cultivated tomato of a pathogen-induced *SpWRKY1* gene from the wild tomato *S. pimpinellifolium* led to a sharp increased resistance to *Phytophthora infestans* when compared with the wild-type plants [80]. The overexpression of *SpWRKY1* was accompanied by the regulation of the expression of an abscisic acid (ABA) biosynthetic gene, which reveals a potentially positive role of *SpWRKY1* in ABA-mediated stomatal closure [80]. Cui et al. [81] state that, amongst the 35 TFs genes from tomato induced by *P. infestans*, the accumulation of the *SpWRKY3* was significantly changed; following a transgenic approach, the overexpression of *SpWRKY3* positively modulated defense response against *P. infestans*, while the resistance was impaired after *SpWRKY3* silencing [81]. These authors state that transgenic tomato plants overexpressing *SpWRKY3* induce the expression of PR genes and reduce ROS accumulation to protect against cell membrane injury, leading to enhanced resistance to *P. infestans*. Following a transcriptomic approach, under the invasion of tomato by *Pseudomonas syringae*, Huang et al. [82] validated the up-regulated expressions of the genes *SlWRKY8*, *SlWRKY23*, *SlWRKY39*, *SlWRKY53*, *SlWRKY80*, and *SlWRKY81*. These authors point to the importance of the functional exploration of tomato WRKYs to provide a subset of candidate target genes for transgenic studies to improve stress tolerance. A tomato line overexpressing *SlWRKY39* already showed enhanced resistance to *P. syringae*, probably via increased the expression of both PR and stress-related genes [83]. WRKY TFs are also involved in tomato defense against root-knot nematodes (RKN). Using microarray analysis, Bhattarai et al. [84] identified the *SlWRKY72a* and *SlWRKY72b* genes as transcriptionally up-regulated during the RKN disease resistance mediated by the R gene *Mi-1*. Silencing of these two genes in tomato resulted in a clear reduction of *Mi-1*-mediated resistance as well as basal defense against RKN. SlWRKY70 was also required for Mi-1-mediated resistance against RKN [97]. Chinnapandi and co-workers [85] observed, in roots overexpressing *SlWRKY45,* enhanced tomato susceptibility to RKN, which was associated with a decreased expression of JA and SA marker genes, proteinase inhibitor and PR protein (PR1), and also the cytokinin response factors CRF1 and CRF6. The Group III WRKY genes *SolyWRKY41*, *SolyWRKY42*, *SolyWRKY53*, *SolyWRKY54*, *SolyWRKY80*, and *SolyWRKY8* were also identified as positive and negative regulators in tomato–*Tomato yellow leaf curly virus* (TYLCV) interaction [86]. It was verified that TFs from Group III were responsive to abiotic and biotic stress, due to the interaction with other proteins, such as mitogen-activated protein kinase 5 (MAPK) and isochorismate synthase (ICS).Additionally, the silencing of *SolyWRKY41* and *SolyWRKY54* decrease accumulation of TYLCV DNA [86].

Regarding NAC TFs, 93 putative NAC proteins were identified in the tomato genome [49] (Table S1). Although NACs were identified in tomato because of their role in diverse developmental processes [98,99], their relevant role in both in abiotic and biotic stress responses is evident [49,53,88,89].

Several studies involving NAC TFs were conducted due to their role in tomato defense by either overexpression and/or silencing, revealing functions as regulators of plant responses to biotic stresses (Table 1). Using an RNA-seq approach, Wang et al. [87] identified a NAC TF-encoding gene (*SlNAP1*), which was strongly induced by several stress conditions. By generating *SlNAP1* transgenic lines and evaluating their responses to biotic stress, these authors verified that *SlNAP1*-overexpressing tomato plants presented a significantly enhanced defense against the bacterial diseases caused by *P. syringae* pv. *tomato* (*Pst*) DC3000, and *Ralstonia solanacearum*. *SlNAP1* was proposed to positively regulate the defense response through the promotion of gibberellins deactivation and by stimulating SA and ABA biosynthesis, further indicating the importance of NAC TFs in crop breeding [87].

Liu and co-authors [88] screened several genes using a VIGS-based approach and found that the severity of the disease caused by *B. cinerea* was increased by the silencing the tomato NAC gene *SlSRN1*. These authors verify a significantly induced expression of *SlSRN1* after infection with *B. cinerea* or *P. syringae* pv. *tomato* (Pst). The expression of the stress related *SlNAC1* gene was also strongly upregulated during *P. syringae* infection, while repression of the *NAC1* ortholog in *Nicotiana benthamiana* resulted in enhanced susceptibility to *Pseudomonas* [89]. Through a yeast (*Saccharomyces cerevisiae*) two-hybrid technology, it was found that *SlNAC1* interact with geminivirus replication enhancer (REn) function from *Tomato leaf curl virus* (TLCV), and overexpression of *SlNAC1* enhances the accumulation of TLCV DNA [90]. 

An interesting finding regarding the role of NAC TFs during pathogen attack is reported by Du and co-authors [100]. Distinct roles were found for two tomato NAC homologues, JA2 (jasmonic acid2) and JA2L(JA2-like), in the regulation of *P. syringae*-triggered stomatal movement; JA2 revealed its positive role in ABA-mediated stomatal closure, whereas JA2L executes stomatal reopening by regulating the expression of genes involved in the metabolism of SA [100].

A total of 167 AP2/ERF TFs were reported in tomato, with all five subfamilies identified (DREB, ERF, AP2, RAV, and Soloist) [101] (Table S1). Within the ERF subfamily, 12 groups were identified [92]. The ERF subfamily is widely involved in the regulation of plant development as well as in responses to abiotic and biotic stresses [92]. Gu and co-authors [102] reported that tomato ERF TFs activate the expression of a wide array of PR genes and play important and distinct roles in plant defense.

The involvement of AP2/ERF TFs (specially belonging to ERF subfamily) in tomato response to pathogens has been the focus of several studies, with reports of altered expression, as well as research involving overexpression and/or silencing of different *AP2/ERF* genes (Table 1). Following a transcriptomic microarray analysis using the necrotrophic pathogen *Alternaria solani*, in a resistant tomato genotype, a high level of expression of the TF *ERF-3* was observed, with *ERF-3* also playing a role on transcription of genes coding for PR-1 [91]. Upadhyay and co-authors [91] point to the involvement of ERF TFs in signaling pathways and defense against necrotrophic pathogens generally mediated through signaling. Amongst the increase of 18 *ERFs* genes post inoculation with *Stemphylium lycopersici,* the positive effect of *ERF2* on tomato resistance to the gray leaf spot disease was highlighted, since, in *ERF2*-silenced plants, the susceptible phenotype was observed after inoculation with *S. lycopersici*, with decreased HR and ROS production [92]. These findings indicate that ERF2 may directly or indirectly regulate PR *Pto* protein kinases, *PR1b1* and *PR-P2* expression, and enhance tomato resistance to *S. lycopersici*. A transcriptomic analysis also revealed 22 AP2/ERF TFs in response to TYLCV infection [101].

The involvement of a AP2-domain TF on the tomato´s defense response to the necrotrophic foliar pathogen *B. cinerea* and the bacterial pathogen *X. campestris* pv. *vesicatoria* was also reported by Buxdorf and co-authors [93]. These authors point the role of the cuticle in plant interactions with pathogens and with their surroundings and the importance of tomato TFs in the regulation of cuticle production. In a *SlSHINE3*-overexpressed line, it was verified resistance to *B. cinerea* infection and to *X. campestris* pv. *vesicatoria*, correlated with cuticle permeability and elevated expression of pathogenesis-related genes *PR1a* and *Allene Oxide Synthase* (*AOS*); on the other hand, the *Slshn3*-silenced line revealed higher susceptibility to *B. cinerea*. 

The *bHLH* gene family in tomato was firstly identified by Sun and co-workers [66]. These authors identified, in the tomato genome, a total of 159 *bHLH* (*SlbHLH*) protein-encoding genes, classified into 21 subfamilies (Table S1). However, there are few studies showing altered expression of tomato *bHLH* genes upon infection of pathogens, as well as research involving the application of genetic engineering tools (Table 1). Kim and Mudgett [71] identified *bHLH132* as highly induced *by X. euvesicatoria* and demonstrate that this TF is induced by microbe-associated molecular patterns (MAMPs) and defense hormones and specifically by *X. euvesicatoria* effector XopD. In this sense, bHLH132 is crucial to the protection of plants against *X. euvesicatoria* infection, playing an important role in tomato immunity [71]. The bHLH MYC2 TF was identified as master regulator in the JA signaling pathway [103]. In fact, it was already verified that the knockout of the tomato *SlMYC2* caused a significant decrease of the expression of the PR genes *SlPR-1* and *SlPR-STH2* and of genes linked to the signaling pathway and JA biosynthesis, besides a decrease on the activities of disease defensive and antioxidant enzymes, with a consequent exacerbation of the *B. cinerea* disease symptoms [94].

In tomato, a genome-wide identification and systematic analyses revealed the existence of 69 *bZIP* genes, classified into 24 distinct subfamilies [75] (Table S1). Li and co-authors [75] observed distinct and diverse expression patterns among the tomato *SlbZIP* genes in different developmental stages and tissues, with several tomato *bZIP* genes possibly involved in responses to different abiotic and biotic stress conditions. The role of the tomato bZIP TF SlAREB1 in response to biotic stress was reported by Orellana et al. [95] (Table 1). When compared with the wild type, mutants overexpressing *SlAREB1* presented an increased tolerance to abiotic stress and a higher expression of genes associated with biotic stress responses (PR proteins, protease inhibitors and catabolic enzymes). These authors hypothesized a potential involvement of SlAREB1 TF in response to pathogens during plant defense [95].

## 5. Final Considerations

TFs, acting through sequence-specific interactions with *cis*-regulatory DNA elements in the promoters of genes, arise as key regulators of tomato defense response against a wide array of pathogens linked to important diseases, together with a complex cross-talk between different signal transduction pathways. Thus, genes that encode TFs are master regulators of stress-related genes and offer extended possibilities related to their use for engineering pathogen resistance in tomato plants, as promising candidates for tomato breeding, taking advantage of molecular techniques that have been recently emerging applied to plant breeding in the genomics and genome editing era.

## Figures and Tables

**Table 1:** Role of transcription factors (TFs) in tomato response to biotic stresses.

TF Family	TF Target Gene	Effect	Method	Ref.
**WRKY**	*SlDRW1*	- Gene expression significantly induced by *Botrytis cinerea*. - Silencing increase severity of disease caused by *B. cinerea.*	VIGS	[79]
	*SpWRKY1*	- Overexpression increase resistance to *Phytophthora infestans.*	Expression vector	[80]
	*SpWRKY3*	- Gene expression significantly induced by *Phytophthora infestans*. - Silencing impaired the resistance to *P. infestans*. - Overexpression increase resistance to *P. infestans.*	VIGS and expression vector	[81]
	*SlWRKY8, SlWRKY23, SlWRKY39, SlWRKY53, SlWRKY80, SlWRKY81*	- Up-regulated genes expression in response to *Pseudomonas syringae* (Pst) pv. tomato DC3000 infection.	-	[82]
	*SlWRKY39*	- Overexpressing increased resistance to *P. syringae.*	Expression vector	[83]
	*SlWRKY72a, SlWRKY72b*	- Up-regulated during root-knot nematodes (RKN) disease resistance mediated by the R gene *Mi-1*. - Silencing resulted in a reduction of Mi-1-mediated resistance and basal defense against RKN.	VIGS	[84]
	*SlWRKY45*	- Overexpression enhanced tomato susceptibility to RKN	Expression vector	[85]
	*SolyWRKY41, SolyWRKY42, SolyWRKY53, SolyWRKY54, SolyWRKY80, SolyWRKY8*	- Genes responsive to *Tomato yellow leaf curly virus* (TYLCV) infection. - Silencing of *SolyWRKY41* and SolyWRKY54 decrease accumulation of TYLCV DNA.	VIGS	[86]
**NAC**	*SlNAP1*	- Overexpressing enhanced defense against Pst DC3000 and *Ralstonia solanacearum.*	Expression vector	[87]
	*SlSRN1*	- Gene expression induced by infection with *B. cinerea* and Pst DC3000. - Silencing increased severity of disease caused by *B. cinerea.*	VIGS	[88]
	*SlNAC1*	- Upregulated gene expression during *Pst* DC3000 infection.	-	[89]
	*SlNAC1*	- Overexpression enhanced the accumulation of *Tomato leaf curl virus* (TLCV) DNA.	Expression vector	[90]
**AP2/ERF**	*ERF-3*	- Upregulated gene expression to *Alternaria solani* infection, using a resistant genotype.	-	[91]
	*ERF-2*	- Silencing revealed aggravated diseases symptoms caused by *Stemphylium lycopersici*.	VIGS	[92]
	*SlSHINE3*	- Silencing revealed higher sensitivity to *B. cinerea*. - Overexpression revealed resistance to *B. cinerea* and to *Xanthomonas campestris* pv. *vesicatoria* infection.	Not referred	[93]
**bHLH**	*bHLH132*	- Transcriptionally highly induced by *Xanthomonas euvesicatoria*. - Silencing enhanced susceptibility to *X. euvesicatoria.*	Expression vector	[71]
	*MYC2*	- Knockout aggravated the *B. cinerea* disease symptoms.	CRISPR/Cas9	[94]
**bZIP**	*SlAREB1*	- Overexpression up-regulate several defense genes associated with biotic stress.	Expression vector	[95]

## Data Availability

Data sharing not applicable.

## Supplementary Materials

The following are available online at https://www.mdpi.com/article/10.3390/biology11020235/s1, Table S1: Number of members of the different families and subfamilies or groups of tomato transcription factors (TFs).
