# Supplementary material for: Defense Strategies: The Role of Transcription Factors in Tomato–Pathogen Interaction
Source: Biology (Basel). 2022 Feb 1;11(2):235. doi: 10.3390/biology11020235 (PMC8869667; doi:10.3390/biology11020235)
Supplement: Supplementary file 1 [file biology-11-00235-s001.zip › biology-1494836-supplementary.pdf]

Table S1 Number of members of the different families and subfamilies or groups of tomato transcription factors (TFs).

TF family	TF subfamily/groups	Nª of members
**WRKY** [96]	Groups I + IIc	33
	Groups IIa + IIb	15
	Groups IId + IIc	23
	Group III	12
	**Total**	**83**
**NAC** [49]	Groups I	20
	Groups IIa	10
	Groups IIb	24
	Groups IIIa	18
	Groups IIIb	21
	**Total**	**93**
**AP2/ERF** [101]	ERF	88
	RAV	3
	AP2	26
	Soliost	1
	DREB	40
	**Total**	**167**
**bHLH** [66]	Group 1	14
	Group 2	5
	Group 3	5
	Group 4	6
	Group 5	11
	Group 6	12
	Group 7	4
	Group 11	7
	Group 13	7
	Group 14	8
	Group 15	11
	Group 16	4
	Group 17	12
	Group 18	3
	Group 19	11
	Group 20	8
	Group 21	9
	**Total**	**159**

TF family (cont.)	TF subfamily/groups (cont.)	Nº of members (cont.)
**bZIP** [75]	BZ1	3
	BZ2	2
	BZ3	4
	BZ4	3
	BZ5	2
	BZ6	4
	BZ7	4
	BZ8	2
	BZ9	2
	BZ10	4
	BZ11	2
	BZ12	2
	BZ13	2
	BZ14	3
	BZ15	7
	BZ16	3
	BZ17	3
	BZ18	1
	BZ19	1
	BZ20	1
	BZ21	1
	BZ22	1
	BZ23	1
	BZ24	12
	**Total**	**69**
